# Hypofractionated stereotactic radiotherapy for brain metastases larger than three centimeters

**DOI:** 10.1186/1748-717X-7-36

**Published:** 2012-03-19

**Authors:** Xue-song Jiang, Jian-ping Xiao, Ye Zhang, Ying-jie Xu, Xiang-pan Li, Xiu-jun Chen, Xiao-dong Huang, Jun-lin Yi, Li Gao, Ye-xiong Li

**Affiliations:** 1 Department of Radiation Oncology, Cancer Hospital & Institute, Chinese Academy of Medical Sciences & Peking Union Medical College, Beijing, China; 2 Department of Radiation Oncology, Jiangsu province cancer hospital, Nanjing, Jiangsu, China

**Keywords:** Brain metastases, Hypofractionated stereotactic radiotherapy (HSRT), Boost

## Abstract

**Background:**

To evaluate the efficacy and outcomes of hypofractionated stereotactic radiotherapy (HSRT) for brain metastases > 3 cm.

**Methods:**

From March 2003 to October 2009, 40 patients with brain metastases larger than 3 cm were treated by HSRT. HSRT was applied in 29 patients for primary treatment and in 11 patients for rescue. Single brain metastasis was detected in 21 patients. Whole brain radiotherapy was incorporated into HSRT in 10 patients for primary treatment. HSRT boosts were applied in 23 patients. The diameters of the brain metastases ranged from 3.1 to 5.5 cm (median, 4.1 cm). The median prescribed dose (not including HSRT boosts) was 40 Gy (range, 20-53 Gy) with a median of 10 fractions (range, 4-15 fractions) to the 90% isodose line. The median dose of the boost was 20 Gy (range, 10-35 Gy) in 4 fractions (range, 2-10 fractions).

**Result:**

The median overall survival time was 15 months. The overall survival and local control rate at 12 months was 55.3% and 94.2%, respectively. Four patients experienced local progression of large brain metastases. Nine patients died of intracranial disease progression. One patient died of radiation necrosis with brain edema.

**Conclusion:**

HSRT was a safe and effective treatment for patients with brain metastases ranged from 3.1 to 5.5 cm. Dose escalation of HSRT boost may improve local control with an acceptable toxicity.

## Background

Twenty to 40% of patients with cancer will develop brain metastases that will result in an impaired quality of life and a reduced survival time [1]. The treatment regimens for brain metastasis include corticosteroids, surgery, whole brain radiotherapy (WBRT), and stereotactic radiosurgery (SRS). The survival of patients with a single metastasis can be prolonged by the combination of surgery and WBRT [2]. For surgically unresectable brain metastases, the combination of SRS and WBRT can prolong the survival of patients with solitary metastases and improve local control in patients with 2 or 3 brain metastases [3]. The prognoses of patients undergoing SRS have been shown to be related to the prescribed dose of the treatment as well as the tumor volume. Low doses and large tumor volumes are adverse factors for local control [4]. Due to the limits of normal tissue tolerance, the Radiation Therapy Oncology Group (RTOG) 90-05 recommended radiation doses of 24 Gy, 18 Gy, and 15 Gy for recurrent brain tumors with maximum diameters of 20 mm or less, 21-31 mm, and 31-40 mm, respectively [5]. Reports have shown that poor prognoses were associated with brain metastases greater than 3 cm in diameter [6]. In recent years, hypofractionated stereotactic radiotherapy (HSRT) has been reported to result in outcomes that are similar to SRS treatment [7-11]. HSRT has a radiobiological advantage over SRS and provides better protection of normal tissues; therefore, HSRT may be a more suitable therapy for large-volume brain metastases. This paper reports preliminary results of a retrospective study of the use of HSRT to treat brain metastases in patients with tumors larger than 3 cm in diameter that was conducted at the Department of Radiation Oncology, Cancer Hospital, Peking Union Medical College, Chinese Academy of Medical Sciences.

## Methods

### Patient data

From May 2003 to October 2009, 40 patients with brain metastases greater than 3 cm in diameter were treated with HSRT. The enrollment criteria included the following: 1, maximum diameter of brain metastasis greater than 30 mm; 2, survival expectancy greater than 3 months; and 3, RPA grade of 1-2 (patients with RPA grade 3 induced by brain metastasis were also enrolled). Of these 40 patients, 29 were treated for primary brain metastases, and 11 were treated for recurrent disease after WBRT. Among the patients undergoing primary treatment, 19 were treated with HSRT alone, and 10 were treated with WBRT plus HSRT. Twenty-one patients had a solitary brain lesion, while 19 patients had 2 to 4 lesions. The pathologies of the primary lesions included 20 patients with non-small-cell lung cancer, 10 patients with small-cell lung cancer, 3 patients with breast cancer, and 7 patients with other cancers. Detailed patient characteristics are summarized in Table 1.

**Table 1:** Patient Characteristics

	Primary treatment with HSRT alone	Primary treatment with WBRT + HSRT	Salvage treatment with HSRT
Total number	19	10	11
Gender			
Male	13	6	5
Female	6	4	6
Median Age, y (range)	55 (38-87)	56 (40-76)	47 (35-73)
≥ 65	6	4	1
< 65	13	6	10
No. of brain metastases			
1	10	4	5
2	9	3	2
3	0	2	4
4	0	1	0
Primary tumor			
Non-small-cell lung cancer	10	6	4
Small-cell lung cancer	2	2	6
Breast cancer	1	2	0
Other	6	0	1
Control of primary tumor			
Controlled	16	6	9
Uncontrolled	3	4	2
Extracranial metastasis			
No	7	2	8
Yes	12	8	3
Karnofsky Performance Score			
≥ 80	10	3	7
< 80	9	7	4
RPA class			
1	4	1	7
2	12	8	4
3	3	1	0
Hypofractionated stereotactic radiotherapy (HSRT) Boost	13	1	9

### Treatment

HSRT was performed in all patients on an outpatient basis. Patients were immobilized in the supine position in a tight thermoplastic stereotactic head mask. Enhanced helical computed tomography (CT) images of 3-mm thickness were obtained using the Novalis™ Brain Scan Treatment Planning System. Gross tumor volume (GTV) was defined by the contrast-enhanced tumor on CT scans with reference to magnetic resonance imaging (MRI) images. Planning target volume (PTV) was defined by adding a margin of 2 mm to the GTV. HSRT was performed using a dynamic conformal arc treatment with a Varian linear accelerator.

The PTV was enclosed by a 90% isodose curve of the prescribed dose in HSRT. The median dose of the first course of HSRT was 40 Gy (range of 20-53 Gy) with a median of 10 fractions(f) (range of 4-15 f). Twenty-three patients were given an HSRT boost 1-3 months after the first course of HSRT. The median dose of the boost was 20 Gy (range, 10-35 Gy) in 4 f (range, 2-10 f). The universal dose fractionation of 40 Gy/20 f or 30 Gy/10 f was used for WBRT. In patients with multiple brain metastases, other metastases were also treated with HSRT simultaneously or sequentially according to position. Detailed HSRT characteristics are summarized in Table 2.

**Table 2:** HSRT Characteristics

	Median (range)
Maximum diameter of brain metastasis (cm)	4.1 (3.1-5.5)
Gross Tumor Volume (GTV) of the first course of HSRT (cm^3^)	17.48 (6.28-64.65)
Dose of the first course of HSRT (Gy)	40 (20-53)
Fractions of the first course of HSRT	10 (4-15)
No. of patients receiving HSRT boost	23
Dose of HSRT boost (Gy)	20 (10-35)
Fractions of HSRT boost	4 (2-10)
GTV of HSRT boost (cm^3^)	10.22 (2.03-37.51)

### Follow-up and statistics

Patients underwent clinical follow-up examinations every 3 months so that we could evaluate their disease status, neurologic symptoms, and performance status. Every patient had at least 1 MRI exam. Each lesion was measured for local tumor response, and tumors were graded according to the following 4 categories: 1) complete remission (CR), indicating the disappearance of all enhanced lesions on MRI; 2) partial remission (PR), indicating evidence of a more than 50% reduction in the cross-sectional dimensions of the tumor on MRI images; 3) progressive disease (PD), indicating a more than 25% increase in size; or 4) stable disease (SD), indicating all other responses. Local tumor control was defined as CR, PR, or SD. Failure to control the local tumor was defined as PD. The appearance of new lesions was defined as intracranial distant metastasis. Survival time was calculated as the time from the beginning of HSRT to follow-up or death. Toxicity was recorded according to the National Cancer Institute Common Terminology Criteria for Adverse Events (CTCAE, version 3.0).

We evaluated local tumor control, intracranial distant metastasis, overall survival, and toxicity as endpoints. Calculations were performed using SPSS 13.0 software in Windows XP (IBM Corporation, Chicago, IL, USA). Survival rate was calculated using the Kaplan-Meier method. Univariate log-rank tests were used to assess the significance of the association between prognostic factors, survival, and local tumor control.

## Results

### Survival and local control

At the time of the last follow-up that was conducted in October of 2010, 8 patients had survived, and 32 were deceased. The median follow-up time of alive patients was 16 months (11-38 months). The median survival time was 15 months [5.5-38 months, 95% Confidence Interval (CI): 10.5-19.5 months]. The mean survival time was 17.8 months (5.5-38 months, 95% CI: 14.6-21.1 months). The overall survival rate at 1, 2, and 3 years was 55.3%, 23.8%, and 15.9%, respectively. Four patients experienced local progression of large lesions. Among these, 2 had been treated for primary brain metastases (1 with HSRT alone and 1 with HSRT plus WBRT), and 2 had undergone salvage treatment. The local control rate at 1, 2, and 3 years was 94.2%, 94.2%, and 78.5%, respectively. Out of the 17 patients who developed new brain metastases, 15 had been treated for primary brain metastases (10 with HSRT alone and 5 with HSRT plus WBRT), and 2 had undergone salvage treatment. Two patients developed local progressions and new brain metastases simultaneously. In patients with multiple metastases, no progression was observed in smaller lesions treated with HSRT simultaneously or sequentially. The intracranial disease-free rate at 1, 2, and 3 years was 64.6%, 41.0%, and 41.0%, respectively.

### Cause of death

Of the 32 patients who died, 12 died of extracranial disease progression, 9 died of intracranial disease progression, 4 died of pulmonary infection, 2 died of depletion, 1 each died of brain edema, secondary malignancy, or complications after craniotomy. The cause of death for the remaining 2 patients was unknown.

### Prognostic factors

The following factors were analyzed in order to determine whether they were related to the prognosis of survival and local tumor control: gender, age, treatment character (primary, salvage), number of brain metastases (1, > 1), status of primary tumor, status of extracranial metastases, Karnofsky performance score (KPS), and RPA class. Controlled primary tumors and KPS scores of 80 or more were advantageous prognostic factors of survival (Table 3). No factor was identified as a significant predictor of local tumor control (Table 4).

**Table 3:** Log-rank Tests for Prognostic Factors Affecting Overall Survival

Factors	*P*-value
Gender	0.077
Age (≥ 65 y vs. < 65 y)	0.238
Treatment character (primary vs. recurrent)	0.725
No. of brain metastases (solitary vs. multiple)	0.189
Primary tumor (controlled vs. uncontrolled)	< 0.001
Extracranial metastasis (yes vs. no)	0.453
KPS (≥ 80 vs. < 80)	0.004
RPA class	0.093

**Table 4:** Log-rank Tests for Prognostic Factors Affecting Local Tumor control

Factors	*P*-value
Gender	0.698
Age (≥ 65 y vs. < 65 y)	0.971
Treatment character (primary vs. recurrent)	0.914
No. of brain metastases (solitary vs. multiple)	0.997
Primary tumor (controlled vs. uncontrolled)	0.394
Extracranial metastasis (yes vs. no)	0.667
KPS (≥ 80 vs. < 80)	0.085
RPA class	0.811

### Toxicity

No acute toxicity was observed. Late toxicity consisting of grade 3-5 brain edema occurred in 5 patients, and it presented as uncontrollable headaches. Four out of 5 of these patients developed new brain metastases and underwent salvage treatment consisting of SRS/HSRT or WBRT. Only 1 patient died of brain edema.

## Discussion

It has been reported that surgery plus WBRT is superior to WBRT alone for patients with solitary brain metastases and good performance statuses [12-14]. However, most patients with brain metastases are not suitable for craniotomies due to the patients' physical statuses, the positions of the metastatic tumor, and the distribution of medical resources. As an alternative to surgery, SRS has been successfully used to treat brain metastasis and generally results in satisfactory local control. Metastatic brain tumors are normally round or oval with little infiltration, making it one of the most suitable targets for SRS. Some studies, including one randomized trial, have shown that, compared to treatment with WBRT alone, SRS plus WBRT resulted in greater improvement in local control of brain metastases and survival times in patients with favorable prognostic factors [2,3].

With SRS treatment, there is a steep dose fall-off in the target region. However, with an increase of target size, the dose gradient decreases. Therefore, while still limited by normal tissue tolerance, the dose of SRS treatment has to decrease with the increase of target size. As a result, the prescribed doses suggested by RTOG 90-05 are 24 Gy, 18 Gy, and 15 Gy for recurrent brain tumors with maximum diameters ≤ 20 mm, 21-31 mm, and 31-40 mm, respectively. It is known that a larger dose is needed to obtain the same tumor control probability for larger tumors. Because of this, large tumor volumes are associated with poor local control in brain metastases [4,6,15].

In recent years, reports have demonstrated that brain metastases treated by HSRT resulted in similar survival durations and long-term toxicities when compared with those treated with only SRS treatments. HSRT is performed with a noninvasive mask and with radiation given in several fractions, which is more comfortable for the patient and has greater advantages with respect to radiobiology and normal tissue protection [16,17]. There is no unified dose fractionation for HSRT. Fahrig et al. compared the fractionations of 5 × 6-7 Gy, 10 × 4 Gy, and 7 × 5 Gy for the treatment of brain metastases and concluded that in cases with brain metastases where the tumor volume is greater than 15 cm^3 ^(diameter > 3 cm) and with regards to toxicity, 10 × 4 Gy was more advantageous than a regimen with less fractionation. However, univariate analysis showed that large tumor volumes are still a disadvantageous prognostic factor [18]. In our study, most of our patients were treated with a fractionation regimen of 10 × 3-5 Gy, and 23 out of 40 patients received an HSRT boost 1-3 months after the first course of HSRT. Only 10% of patients (4 out of 40) were found to have local recurrence. In fact, the local control rate (94.2% at 1 year) was comparable to other studies of SRS and HSRT treatments. Therefore, we concluded that brain metastasis with tumors > 3 cm can be well controlled with higher doses of radiation. Patients in our study had a median survival time of 15 months, which was greater than that of most other reports of SRS/HSRT treatments [8-11,17,19]. Though this may be a result of patient selection, our study also revealed that local control may translate to survival advantages in patients with metastatic brain tumors > 3 cm.

The univariate analysis showed that a controlled primary tumor and a good performance score were associated with better survival. However, contrary to previous studies, our data showed that RPA class was not a significant factor predicting survival time [20,21]. RPA class was not found to be relevant with survival probably because of the small number of patients. No prognostic factors, including tumor volume, were found to be relevant with respect to local tumor control. This suggests that dose escalation could be used to decrease the harmful effects of large tumor volume on local control.

Five patients developed serious late toxicities that presented as brain edema. Four of them, however, also had new brain metastases and underwent salvage treatment. Only 1 patient definitely died of brain edema and necrosis was displayed on MRI exams, and there was no evidence of intracranial recurrence in this patient. This patient had a brain metastasis in left temporal lobe from lung adenocarcinoma. She was first arranged WBRT with a dose fractionation of 40 Gy/15 f. Four months after WBRT, symptoms recrudesced and recurrence was detected On MRI. Then HSRT with a dose fractionation of 27 Gy/9f and boost of 24 Gy/3f was prescribed to the recurrent lesion. Eight months later, she developed epilepsy and edema with necrosis was displayed on MRI. The patient refused further treatment including cerebral ventricular shunt. Finally she died 12 months after the performance of HSRT with no evidence of tumor progression. Though in some cases it was difficult to determine the cause of the brain edema when suspicious recurrence existed, her death was deemed as treatment related. Nevertheless, she gained 12 months survival time from brain metastasis recurrence and we supposed the edema may be released by cerebral ventricular shunt if there was no tumor progression. So the toxicity was deemed as acceptable concerning good local control.

The diameters of the brain metastases in this study ranged from 3.1 to 5.5 cm (median, 4.1 cm). It was unknown whether HSRT was suitable for bigger lesions beyond this range. Since large brain metastases have mass effect and normally induce obvious brain edema, we recommend HSRT should be arranged with cautions in case of brain metastases > 5.5 cm.

## Conclusion

In conclusion, we found that HSRT was a safe and effective therapeutic option for patients with brain metastases ranged form 3.1 to 5.5 cm. Dose escalation of the HSRT boost after the first course of HSRT when many tumors were reduced in volume may improve local control, while the dose was still within the limits of acceptable toxicity. Due to the small sample size in this study, further research is needed.

## Abbreviations

HSRT: hypofractionated stereotactic radiotherapy; WBRT: whole brain radiotherapy; SRS: stereotactic radiosurgery; RTOG: Therapy Oncology Group; RPA: recursive partitioning analysis; CT: computed tomography; GTV: gross tumor volume; MRI: magnetic resonance imaging; PTV: planning target volume; F: fraction; CR: complete remission; PR: partial remission; PD: progressive disease; SD: stable disease; CTCAE: common terminology criteria for adverse events.

## Competing interests

We have no personal or financial conflicts of interest, and have not entered into any agreement that could interfere with our access to the data on the research, or upon our ability to analyze the data independently, to prepare manuscripts, and to publish them.

## Authors' contributions

JPX provided the concept of the present study; XSJ and JPX contributed to the collection and analysis of the data, wrote the manuscript; YJX made the treatment planning; YZ, XPL, XJC, XDH, JLY and LG selected the patients; YXL and JPX gave the administrative support and the final approve of the manuscript to be published. All authors have read and approved the final manuscript.
